# Bioconversion of Tyrosine- and Tryptophan-Derived Biogenic Amines by Neuropathogenic Bacteria

**DOI:** 10.3390/biom8010010

**Published:** 2018-02-13

**Authors:** Aneela Taj, Nusrat Jamil

**Affiliations:** Department of Microbiology, University of Karachi, Karachi 75270, Pakistan; ain2005_ku@yahoo.com

**Keywords:** biogenic amines, dopamine degradation, physiology, neuropathogenic bacteria, neurochemicals, high performance liquid chromatography with electrochemical detection (HPLC-EC)

## Abstract

The biochemical potential of pathogenic bacteria may cause alteration in the neurophysiological environment; consequently, neuroendocrine and immune responses of the host are modulated by endogenously produced metabolic products of neuropathogenic bacteria. The present study was designed to detect the derived biogenic amines in spent culture media of *Bacillus cereus* (Bc), *Clostridium tetani* (Ct), *Listeria monocytogenes* (Lm), and *Neisseria meningitidis* (Nm). Overnight grown culture in different culture media i.e., Nutrient broth (NB), Luria basal broth (LB), Brain Heart Infusion broth (BHI), and human serum supplemented RPMI 1640 medium (RPMI) were used to prepare filter-sterilized, cell-free cultural broths (SCFBs) and subjected to high performance liquid chromatography with electrochemical detection (HPLC-EC) along with the control SCFBs. Comparative analysis of biogenic amines in neuropathogenic bacterial SCFBs with their respective control (SCFB) revealed the complete degradation of dopamine (DA) into its metabolic products by Bc, Ct, and Nm, whereas Lm showed negligible degradation of DA. A relatively high concentration of 5-hydroxyindol acetic acid (5HIAA) by Bc in NB and LB indicated the tryptophan metabolism by the serotonin (5HT) pathway. Our study suggests that microbial endocrinology could help unravel new perspectives to the progression of infectious diseases.

## 1. Introduction

Microbial endocrinology refers to the ability of microorganisms to produce, recognize, and respond to neurochemicals that originate either in within these microorganisms themselves or within the host they inhabit [1,2]. In addition, the term neurochemical defines any chemical produced by microorganisms that is equally recognized and functions as a neurotransmitter and/or neuromodulator and neurohormone within the mammalian host system [2,3]. These neurochemicals are mainly low molecular-weight, first-messenger molecules that include amino acids and amino acid derivatives i.e., catecholamine (dopamine (DA)) and indols (serotonin (5HT)) and their respective downstream products. Therefore, neurochemicals are pivotal for cell–cell signaling, which is essential for the normal functioning of the nervous, endocrine, immune, and neuroimmune systems of the host [4]. 

It is perhaps a well-established fact that microorganisms have evolved specific systems for sensing these neurochemicals produced within the host during the physiological response to infection-mediated stress [2,5,6]. Furthermore, neurochemicals can be exploited by infectious microbes as an environmental signal to initiate cellular processes, including growth, and the elaboration of virulence-associated factors leading to the activation of pathogenic processes, which ultimately results in the progression of infection [5,6,7,8,9,10,11,12,13].

An extensive literature search revealed that experimental investigations focusing on neuroactive neurochemicals have been confined to bacteria, mainly on *Escherichia coli* O157:H7, *E. coli* K-12, *Pseudomonas aeruginosa*, *Salmonella enterica*, *Yersinia enterocolitica*, *Myxobacterium polyangium*, *Streptococcus faecalis*, *Bacillus cereus*, lactic acid bacteria, bifidobacteria, and gut microbiota [8,9,10,11,12,13,14,15,16,17,18,19,20,21,22], as well as a few eukaryotic microorganisms i.e., *Rhodospirillum rubrum*, *Candida guilliermondii*, and *Saccharomyces cerevisiae* [15,16,17]. However, no consideration has been given to the neuropathogenic microbes which are at present known producers of oxidative stress in a host during the infection course, thus affecting cardinal responses of the host [23,24,25,26,27,28,29]. Therefore, contrary to previous research, we focused on screening the biochemical potentials of neuropathogenic bacteria expressed in response to neurochemicals provided in growth media as nutrients. 

Most of the media used today, especially enriched media, are largely of animal origin and contain highly undefined source materials as nutrients i.e., brains, hearts, and yeast of mammalian and eukaryotic origin, respectively [2,30]. Thus, this provides strong evidence that microbiological media may contain variable amounts of substrates, cofactors [2], and amino acid precursor molecules that can be used by these neuropathogens to either produce or convert provided nutrients into neuroactive neurochemicals that may significantly interfere with the host neurophysiological system. 

Therefore, the present study was designed to investigate the biochemical potentials of neuropathogenic bacteria grown in microbiological media i.e., Luria basal broth (LB), Nutrient broth (NB), Brain Heart Infusion broth (BHI), and human serum-supplemented RPMI 1640 medium (RPMI) containing nutrients of diversified origin. In particular, biogenic amine-derived conversion was investigated in spent culture media of *B. cereus* (Bc), *Clostridium tetani* (Ct), *Listeria monocytogenes* (Lm), and *Neisseria meningitidis* (Nm).

## 2. Results

### 2.1. Quantification of Dopamine

**Figure 1:**
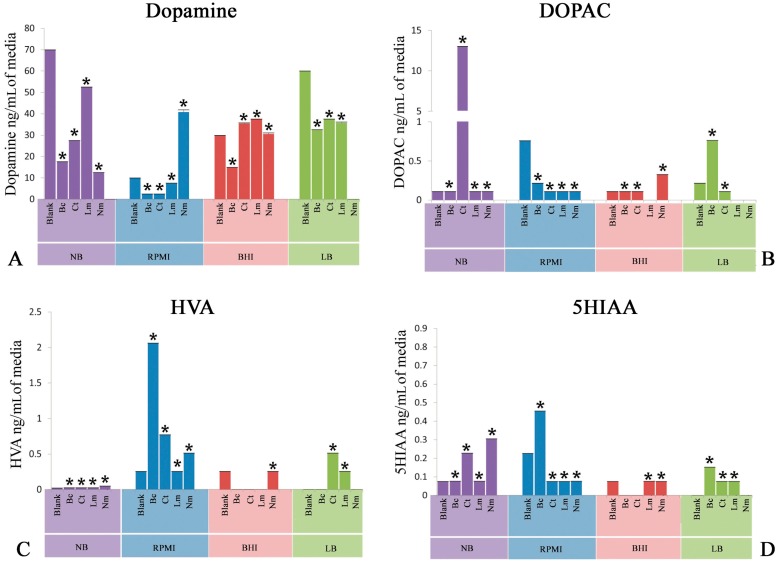
Quantification of neuroactive neurochemicals in spent culture media post inoculation of *Bacillus cereus* (Bc), *Clostridium tetani* (Ct), *Listeria monocytogenes* (Lm), and *Neisseria meningitides* (Nm) as well as blank media (Control). (**A**) Levels of dopamine in Luria basal broth (LB), Nutrient broth (NB), brain heart infusion broth (BHI), and human serum-supplemented RPMI 1640 medium (RPMI); (**B**) Levels of 3,4-dihydroxyphenylacetic acid (DOPAC) in LB, NB, BHI, and RPMI; (**C**) Levels of homovanillic acid (HVA) in LB, NB, BHI, and RPMI; and (**D**) Levels of 5-hydroxyindoleacetic acid (5HIAA) in LB, NB, BHI, and RPMI. All spent media were compared with their respective blank media (Control). Values are expressed as means ± standard error of means (SEMs). * *p* < 0.05.

This experiment was delineated to evaluate the bioconversion of tyrosine amino acid to neuroactive neurochemical i.e., dopamine (DA) provided in different nutritional models consisting of LB, NB, BHI, and PRMI media by neuropathogenic bacteria. Figure 1 depicts the high performance liquid chromatography with electrochemical detection (HPLC-EC) quantification profile of DA. It is noteworthy to mention herein that Bc significantly (*p* < 0.05) utilized tyrosine and converted into DA in all the nutritional background. Ct, in contrast, yielded a significant (*p* < 0.05) amount of DA in all nutritional backgrounds. Furthermore, Lm showed a slight conversion (*p* < 0.05) of tyrosine into DA. On the other hand, Nm revealed a significant (*p* < 0.05) production of DA in all media used in present study.

### 2.2. Quantification of 3,4-Dihydroxyphenylacetic Acid 

Another set of experiments was conducted to detect the bioconversion of DA degradation intermediate 3,4-dihydroxyphenylacetic acid (DOPAC). It is significant to note that all the neuropathogenic bacteria i.e., Bc, Ct, Lm, and Nm significantly (*p* < 0.05) produced this intermediate in all nutritional models. In addition, quantification of DOPAC in Ct SCFB prepared in NB revealed significant (*p* < 0.05) bioconversion (Figure 1B).

### 2.3. Quantification of Homovanillic Acid

Next, the evaluation of homovanillic acid (HVA) was carried out in nutritional model of spent bacterial cultures of Bc, Ct, Lm, and Nm. Bc significantly carried out the downstream degradation of DA and produced a significant (*p* < 0.05) amount of HVA in all media (Figure 1C). Ct, on the other hand, showed significant (*p* < 0.05) conversion of tyrosine into HVA in all media except in BHI, which showed a negligible amount of HVA (Figure 1C). Moreover, Nm showed a profound yield (*p* < 0.05) of HVA in NB, whereas the rest of the media were found yield a moderate conversion (*p* < 0.05) of this neuroactive neurochemical as compared to NB. In addition, Lm showed moderate production (*p* < 0.05) of HVA in all nutritional models. These results highlight that the complete degradation of DA into HVA is not possible in Lm.

### 2.4. Quantification of 5-Hydroxyindoleacetic Acid 

In this set of experiments, tryptophan-derived bioconversion by neuropathogens was evaluated through the quantification of 5-hydroxyindoleacetic acid (5HIAA) using HPLC-EC. It is significant to note that Bc significantly (*p* < 0.05) converted this precursor amino acid into 5HIAA in the nutritional background of RPMI only, while a negligible amount of 5HIAA was detected in the rest of the nutritional models (Figure 1D). In contrast, Ct spent nutritional models of RPMI and LB significantly (*p* < 0.05) yielded the production of 5HIAA (Figure 1D). Furthermore, quantification of 5HIAA in spent media of Nm revealed that this bacterium was found to be capable of carrying out the significant bioconversion (*p* < 0.05) in all three media except LB. Finally, Lm showed significant (*p* < 0.05) bioconversion of tryptophan into 5HIAA in the nutritional background of RPMI and LB (Figure 1D). 

## 3. Discussion

During the course of infections, bacteria may also influence the metabolic machinery and/or processes of the host. Amino acids serve as bacterial growth factors. Bioconversion of these amino acids may yield some bioactive molecules of neurological significance for e.g., DA and 5HT. Tyrosine and tryptophan are the main precursors of the above mentioned neurotransmitters. Therefore, the present study was designed to detect the biogenic amines-derived conversion of the provided nutrients in spent bacterial culture media of different compositions by HPLC-EC. 

The present study demonstrated that media composition has profound effects on the biochemical potentials of neuropathogenic bacteria. NB contains beef extract, while beef’s heart and brain infusions are the main constituents of BHI; hence, both may mimic the animal milieu. On the other hand, LB is mainly composed of yeast extract and RPMI is supplemented with human serum, providing yeast and human biochemical backgrounds, respectively. Surprisingly, a comparative analysis of blank media with spent media indicated that extensive degradation of DA was found in all of these bacterial spent media i.e., *Cl. tetani*, *B. cereus*, and *N. meningitides*, except *L. monocytogenes* only negligibly degraded the DA (Figure 1). 

**Figure 2:**
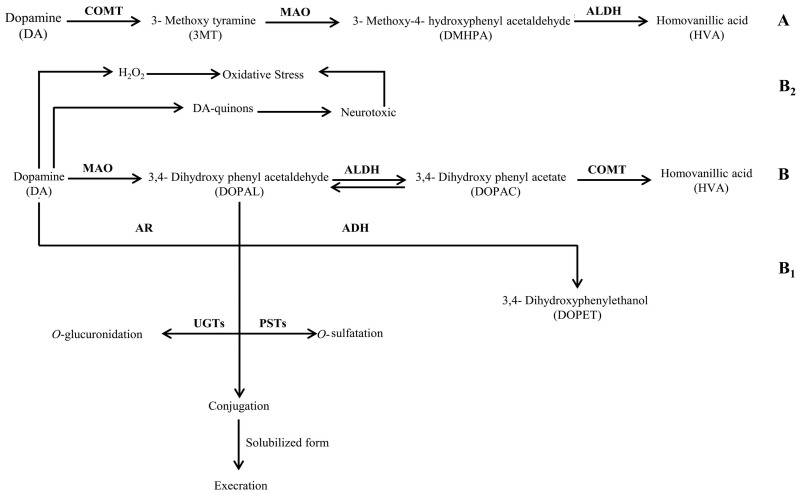
Schematic representation of the chemical pathway for the possible utilization of dopamine by neuropathogenic bacteria. Enzymes involved are: ALDH: aldehyde dehydrogenase; AR: aldehyde reductase; ADH: alcohol dehydrogenase; COMT: catechol-*O*-methyl transferase; MAO: monoamine oxidase; PSTs: phenolsulfotransferases; and UGTs: uridine diphosphoglucuronosyltransferases.

It is a well-documented fact that the enzymatic degradation of DA leads to the formation of the main end product i.e., HVA. This metabolite is produced from intermediate products i.e., DOPAC and 3,4-dihydroxy phenyl acetaldehyde (DOPAL) through irreversible reactions as illustrated in Figure 2—pathway B. However, the conversion of DOPAL into DOPAC [31] is via a reversible reaction; therefore, rapid interconversion of both intermediate metabolites may be possible. Although we did not check the presence of DOPAL, the presence of a relatively lower quantity of DA in the control and an increased quantity of DOPAC in the spent media models reflected that the three bacteria investigated in our study i.e., Bc, Ct, and Nm extensively degraded DA via pathway B (Figure 2). Thus, it can be speculated from the results of the present study that the metabolic activity of Bc, Nm, and Ct may lead to the development of symptoms such as altered mental status, fever, muscle and joints pain, stiff neck, and spasms. These symptoms mainly develop during the active state of infection caused by these bacteria. Nonetheless, the metabolic potentials of these bacteria, i.e., the rapid downstream conversion of DA found in the present study, may serve as a contributory factor to the development of the symptoms of infection. Interestingly, all of the abovementioned symptoms are similar to those of Parkinson’s disease (PD), a well-studied neurodegenerative disease that is mainly associated with the loss of dopaminergic neurons due to the rapid depletion of DA [32]. Moreover, accumulating evidence suggests that many microbes and their components, i.e., lipopolysaccharides, are associated with the development of PD-like symptoms in the host during infection [33,34,35,36]. Therefore, the results of the present study take these findings a step further and provide strong evidence that neuropathogenic bacteria are capable of degrading the available DA in a mammalian host’s blood and peripheral nervous system, thus contributing to the development of PD-like symptoms and repercussions of the infections caused by these bacteria. 

The results of the present study can be further explained by the fact that Bc, Ct, and Nm may follow pathway B_2_ (Figure 2) for the oxidative deamination of DA that may generate increased amounts of hydrogen peroxide (H_2_O_2_), causing oxidative stress in dopaminergic neurons. Elevated enzymatic oxidation of DA by a metal-catalyst (Fe^3+^), on the other hand, may result in the production of DA-quinons that can react further on to form a variety of neurotoxic compounds and protein adducts. DA may further undergo the conjugation reaction via *O*-glucuronidation and *O*-sulfatation, as illustrated in Figure 2—pathway B_1_, both in central nervous system and in the periphery before excretion [31]. Consequently, neuronal cell damage and even neurodegeneration may occur, contributing to the progression of infection in the host when infected by any of these bacteria. Presumably, detection of these conjugates in the urine may not only give insight to the ongoing scenario, but also be one of the diagnostic tools to differentiate between infections and other physiological manifestations. In contrast, the absence of DOPAC in all spent media of Lm indicated that Lm followed the same pathway B (Figure 2) for the negligible degradation of DA. These values demonstrated the fact that the further conversion of DOPAC into HVA is completely eliminated in a Listerial biochemical background. However, the excessive amount of DA is associated with the impaired enzymatic degradation of DA. Another possible reason for the elevated amount of DA may presumably be the intervention of Lm in the uptake of DA by presynaptic neurons. The vesicular monoamine transporter 2 (VMAT2) may be affected by Lm. Furthermore, detection of HVA exclusively in the LB spent medium of Lm presumably reflected the yeast extract enzymatic support which facilitated the bioconversion of DA into HVA by Lm. 

**Figure 3:**
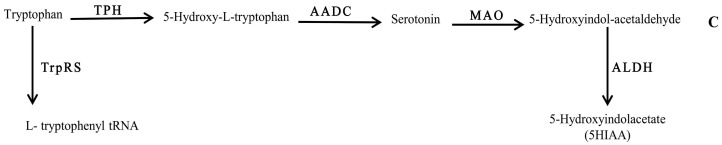
Schematic representation of the chemical pathway for the possible utilization of serotonin by neuropathogenic bacteria. Enzymes involved are: TPH: tryptophan 5-monoxygenase; AADC: aromatic l-amino acid decarboxylase; MAO: monoamine oxidase ALDH: aldehyde dehydrogenase; and TrpRs: tryptophan–tRNA ligase.

Tryptophan metabolism, on the other hand, was found to be influenced by the compositional background of the bacteria. Interestingly, detection of 5HIAA in RPMI spent medium of Bc (Figure 3) highlighted the tryptophan bioconversion of this bacterium, which may have occurred via pathway C (Figure 3). Therefore, it can be assumed that the biochemistry of 5HT is strongly affected in bacterial infections. 

Much evidence has suggested the existence of complete biosynthetic pathways for the catecholamines [2,4] and the presence of key enzymes, i.e., monoamine oxidase, catechol *O*-methyltransferase, and tryptophan hydroxylase, that are involved in the metabolism of neuroactive neurochemicals [2] in bacteria. Furthermore, a growing number of studies have reported the identification of serotonin and catecholamines in microbes [2,37,38,39,40,41] that may be used by them to interact with a mammalian host. Therefore, the present findings significantly contribute to giving a clearer picture of how neuropathogenic bacteria can actively use and/or convert the neurohormonal products of the host for their own advantage, thus resulting in the progression of infection.

It is important to note that during the course of bacterial growth, bacteria metabolize the available molecules. Therefore, in the present study, we selected the model of media composition that better mimics the human cellular background. Lm and Nm are the group of pathogens that irrespective of the cellular background give the similar cellular yield. Although these results were shown in an in vitro environment, the manifestations may also be applicable in in vivo conditions. Therefore, we propose that during the infections, bacteria could influence the yield of biogenic amines in the infected host. Although these degradation pathways are well documented, we further speculate that bacterial growth inside the host will be active through these pathways. Moreover, neuropathogenic bacteria are capable of intervening in host biogenic amines metabolic pathways, thus affecting host physiology. Consequently, this may implicate their effects on host cardinal functions i.e., neurotransmission, behavior [42], memory, and cognition, as well as peripheral and neuroimmune functions, which significantly contribute to disease progression.

## 4. Materials and Methods

### 4.1. Chemicals

Unless otherwise stated, all the analytical grade chemicals and formulated bacteriological media were purchased from Merck, Frankfurter, Darmstadt, Germany. Neurochemical standards were obtained from Sigma, St. Louis, MO, USA. HPLC-EC system with Chromeleon v.6.8.0 software was acquired from Merck Frankfurter.

### 4.2. Bacteria

The study reported herein was carried out with neuropathogens of bacterial etiology i.e., *B. cereus*, *Cl. tetani*, *N. meningitides*, and *L. monocytogenes* isolated and identified in our lab during another study reported elsewhere [43]. 

### 4.3. Microbiological Media

Bacteriological growth media varying from simple to enriched nutrients were selected and used for conducting these experiments. In particular, LB, NB, BHI, and RPMI were used for the growth of neuropathogenic bacteria (Table 1). These media contain the nutrients of mammalian and eukaryotic origin.

### 4.4. Experimental Procedure

In order to prepare filter-sterilized, cell-free cultural broth (SCFB), all of the abovementioned bacterial species, i.e., Lm, Nm, Ct, and Bc, were grown separately to give confluent growth via inoculating a single colony of each in 100 mL of NB, LB, BHI, and RPMI (10 mL) for 24 h at 37 °C. Anaerobic conditions were maintained by providing 5% CO_2_. After incubation, each of the cultural broth samples was centrifuged at 3200 g for 10 min; pellet was discarded and each supernatant was separately air-dried overnight at ambient temperature. Dried broth was scraped off by adding 2 mL of distilled water Each SCFB was filter-sterilized using a 0.2-micron membrane Millipore filter. SCFBs were marked as Lm, Nm, Ct, and Bc. Blank broth i.e., without bacterial inoculation of each of the above mentioned media, were air-dried and filter-sterilized as well, to serve as control SCFBs. All of the experimental procedures were run in triplicate.

### 4.5. Sterility Check

Sterility of all the SCFBs was determined prior to HPLC-EC. Ten microliters of each SCFB was separately streaked on nutrient agar plates. SCFBs that did not yield any bacterial growth after overnight incubation at 37 °C were used for carrying out experiments. Sterility was maintained throughout this study. 

### 4.6. HPLC-EC 

The HPLC-EC system consisted of a Dionex ED50 electrochemical detector, a glassy carbon working electrode, 5 μm octadeclysilane (ODS) reverse phase, 250 × 4.6 mm, C-18 column, and Chromeleon v.6.8.0 software. The column buffer was consisted of 0.1 M NaH_2_PO_4_, 0.1% octyl sodium sulphate, 0.0035% ethylenediaminetetraacetic acid (EDTA), and 14% methanol (pH 2.9 adjusted with phosphoric acid). The flow rate of the pump (Dionex Ultimate 3000) was 1 mL/min. The sensitivity of the detector was 1.0 nA and the potential of the working electrode was 0.6 V. Twenty-five microliters of supernatant was injected into the HPLC-EC system. The peak areas generated from the supernatants were recorded and quantified by comparison with external standards containing 100 ng/mL of DA, DOPAC, HVA, 5HIAA, and 200 ng/mL of 5HT.

### 4.7. Statistics

The significance (*p* < 0.05) of between media group differences was analyzed using one way ANOVA in SPSS version 16, SPSS Inc. Released 2007. SPSS for Windows, Version 16.0. Chicago, SPSS Inc., Chicago, IL, USA. Statistical data are expressed as means ± standard error of means (SEMs).

## Figures and Tables

**Table 1:** Description of the experimental challenges used in the current study.

Serial Number	Bacteria	Media Used for Filter- Sterilized Cell-Free Cultural Broths (SCFBs) Preparation
01	*Cl. tetani*	Nutrient Broth
		Luria Basal Broth
		Brain Heart Infusion Broth
		RPMI 1640 with human serum
02	*L. monocytogenes*	Nutrient Broth
		Luria Basal Broth
		Brain Heart Infusion Broth
		RPMI 1640 with human serum
03	*B. cereus*	Nutrient Broth
		Luria Basal Broth
		Brain Heart Infusion Broth
		RPMI 1640 with human serum
04	*N. meningitides*	Nutrient Broth
		Luria Basal Broth
		Brain Heart Infusion Broth
		RPMI 1640 with human serum
05	Control	Blank Nutrient Broth
		Blank Luria Basal Broth
		Blank Brain Heart Infusion Broth
		Blank RPMI 1640 with human serum
